# *MYBPC1*, an Emerging Myopathic Gene: What We Know and What We Need to Learn

**DOI:** 10.3389/fphys.2016.00410

**Published:** 2016-09-14

**Authors:** Janelle Geist, Aikaterini Kontrogianni-Konstantopoulos

**Affiliations:** Department of Biochemistry and Molecular Biology, University of Maryland School of MedicineBaltimore, MD, USA

**Keywords:** MyBP-C slow, *MYBPC1*, actomyosin crossbridges, phosphorylation, distal arthrogryposis myopathy

## Abstract

Myosin Binding Protein-C (MyBP-C) comprises a family of accessory proteins that includes the cardiac, slow skeletal, and fast skeletal isoforms. The three isoforms share structural and sequence homology, and localize at the C-zone of the sarcomeric A-band where they interact with thick and thin filaments to regulate the cycling of actomyosin crossbridges. The cardiac isoform, encoded by *MYBPC3*, has been extensively studied over the last several decades due to its high mutational rate in congenital hypertrophic and dilated cardiomyopathy. It is only recently, however, that the *MYBPC1* gene encoding the slow skeletal isoform (sMyBP-C) has gained attention. Accordingly, during the last 5 years it has been shown that *MYBPC1* undergoes extensive exon shuffling resulting in the generation of multiple slow variants, which are co-expressed in different combinations and amounts in both slow and fast skeletal muscles. The sMyBP-C variants are subjected to PKA- and PKC-mediated phosphorylation in constitutive and alternatively spliced sites. More importantly, missense, and nonsense mutations in *MYBPC1* have been directly linked with the development of severe and lethal forms of distal arthrogryposis myopathy and muscle tremors. Currently, there is no mammalian animal model of sMyBP-C, but new technologies including CRISPR/Cas9 and xenografting of human biopsies into immunodeficient mice could provide unique ways to study the regulation and roles of sMyBP-C in health and disease.

## Introduction

**Figure 1:**
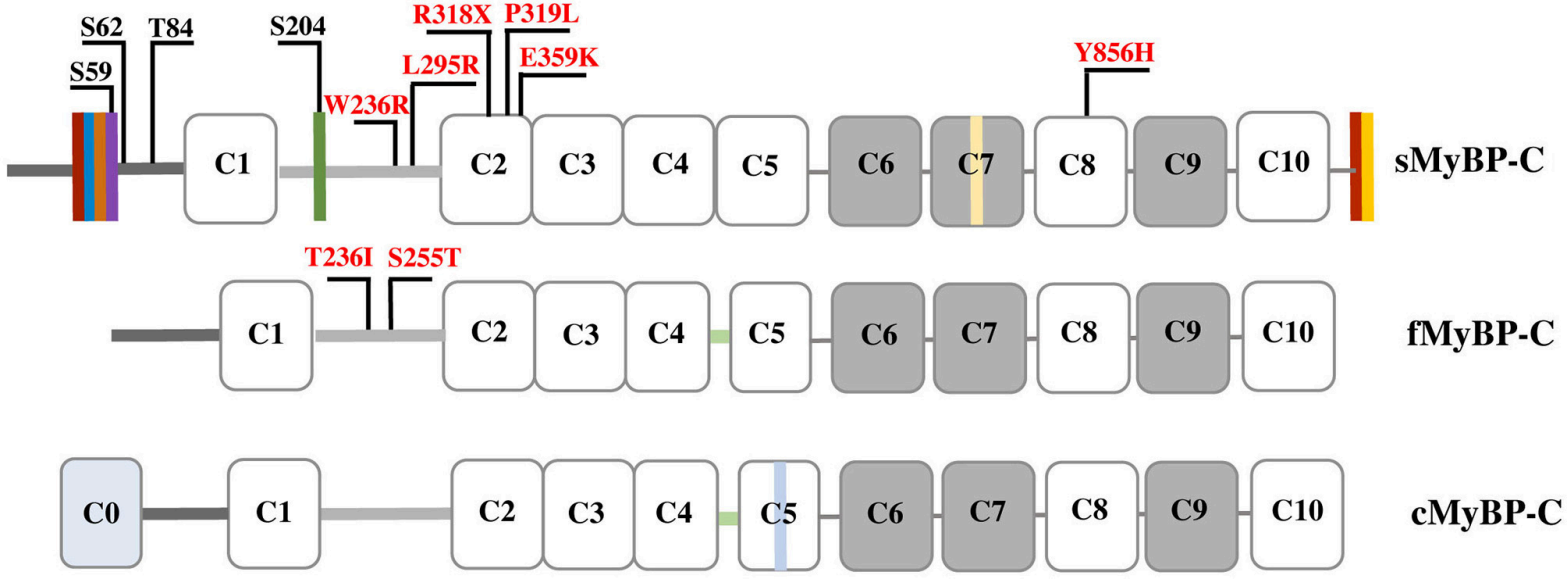
**Schematic representation of the three MyBP-C isoforms**. Dark and light gray lines correspond to the Pro/Ala-rich region and the M-motif, respectively, while white and gray rectangles represent Ig and Fn-III domains, respectively. Vertical colored boxes in the Pro/Ala-rich region, the M-motif, Fn-III domain C7 and the extreme COOH-terminus of sMyBP-C indicate alternative spliced segments. fMyBP-C and cMyBP-C share a conserved linker region between C4 and C5, denoted in light green. cMyBP-C contains an additional Ig domain, C0, and an isoform-specific insertion in C5 shown in light blue. Phosphorylation sites in the Pro/Ala-rich region and the M-motif of sMyBP-C are indicated in black, and myopathic mutations in sMyBP-C and fMyBP-C in the M-motif and Ig domains C2 and C8 are shown in red.

Myosin Binding Protein-C (MyBP-C) comprises a family of accessory proteins expressed in striated muscles, and constitutes 2–4% of the myofibrillar protein mass (Okagaki et al., 1993; Moss et al., 2015) There are three MyBP-C isoforms encoded by different genes; slow (s) skeletal MyBP-C is encoded by *MYBPC1* present in human chromosome 12, fast (f) skeletal MyBP-C is encoded by *MYBPC2* present in human chromosome 19, and cardiac (c) MyBP-C is encoded by *MYBPC3* present in human chromosome 11 (Weber et al., 1993). While cMyBP-C is selectively expressed in cardiac muscle, fMyBP-C and sMyBP-C co-exist in fast and slow twitch muscles at varying amounts (Ackermann and Kontrogianni-Konstantopoulos, 2011b). The three isoforms share structural and sequence homology, primarily consisting of immunoglobulin (Ig), and fibronectin-III (Fn-III) domains, referred from the NH_2_-terminus to the COOH-terminus as C1-C10; notably, the cardiac isoform contains an additional Ig domain, termed C0 (Figure 1; Ackermann and Kontrogianni-Konstantopoulos, 2010).

MyBP-C interacts directly with both thick and thin filaments via its NH_2_- and COOH-termini. The NH_2_-terminal domains C1-M-C2 bind to myosin subfragment 2 (S2) (Gruen et al., 1999), while the COOH-terminal C10 domain binds to light meromyosin (LMM) (Okagaki et al., 1993; Harris et al., 2011). The latter interaction is enhanced by binding of domains C8-C10 to titin that is likely instrumental in the periodic arrangement of MyBP-C in the C-zone (Craig and Offer, 1976; Freiburg and Gautel, 1996; Luther et al., 2008; Luther and Craig, 2011). In addition to binding to S2, the NH_2_-terminal C0-C1-M-C2 region contains (relatively weak) binding sites for actin (Kulikovskaya et al., 2003; Whitten et al., 2008; Ackermann et al., 2009; Shaffer et al., 2009; Orlova et al., 2011; Bhuiyan et al., 2012), although recently domains C6-C10 were suggested to mediate high-affinity binding to actin (Rybakova et al., 2011). The interactions at the NH_2_-terminus of MyBP-C are highly dynamic and regulated via phosphorylation (Figure 1; Gruen et al., 1999; Sadayappan et al., 2005; Shaffer et al., 2009; Barefield and Sadayappan, 2010). Accordingly, phosphorylation of cMyBP-C within the M-motif accelerates contraction by disrupting binding to myosin, thereby increasing the probability of binding to actin and thus the rate of force development (Sadayappan and de Tombe, 2012; Walcott et al., 2015; Colson et al., 2016; Moss, 2016; Previs et al., 2016).

Although several questions remain unanswered regarding the (patho)physiology of MyBP-C, early and recent work (primarily on the cardiac isoform) has postulated that through its direct binding to both actin and myosin filaments, it contributes to their assembly and stabilization, modulates the cycling of actomyosin crossbridges, and regulates the ATPase activity of myosin (Martyn, 2004; McClellan et al., 2004; de Tombe, 2006; Oakley et al., 2007; Ackermann and Kontrogianni-Konstantopoulos, 2010, 2013; James and Robbins, 2011; Rybakova et al., 2011; Ackermann et al., 2013). Below, we summarize old and new information on slow skeletal MyBP-C, highlight its direct involvement in disease pathogenesis and provide a perspective on its roles and regulation.

## sMyBP-C: a complex sub-family of proteins regulated by phosphorylation

MyBP-C proteins are highly modular consisting of tandem immunoglobulin (Ig) and fibronectin-III (Fn-III) modules interspersed with unique short amino acid segments (Figure 1; Winegrad, 1999). Ig domain C1 is flanked by a ~50 amino acids long Proline/Alanine-rich motif (Pro/Ala-rich motif) and a ~100 amino acids long MyBP-C specific motif, referred to as M-motif (Craig et al., 2014). Single transcripts have been identified for the mammalian cardiac and fast isoforms, which result in proteins of ~140 and ~130 kDa, respectively (Yasuda et al., 1995). sMyBP-C is unique, however, as there are several mammalian variants that have been characterized ranging in size from 126 to 131 kDa (Ackermann et al., 2015b). This size variability results from extensive splicing of small amino acid segments within the Pro/Ala-rich motif, the M-motif, Fn-III domain C7, and the extreme COOH-terminus (Ackermann and Kontrogianni-Konstantopoulos, 2011b). The different sMyBP-C variants are co-expressed in different amounts and combinations in both slow- and fast-twitch skeletal muscles were they co-exist with fMyBP-C (Ackermann and Kontrogianni-Konstantopoulos, 2011b, 2013).

Phosphorylation of cMyBP-C contributes significantly to contractile regulation (Sadayappan et al., 2005; Stelzer et al., 2006; Gresham et al., 2014; Gupta and Robbins, 2014; Gresham and Stelzer, 2016; Mamidi et al., 2016; Moss, 2016; Previs et al., 2016). Contrary to early studies suggesting that sMyBP-C is not subjected to phosphorylation (Gruen et al., 1999), work from our group demonstrated that similar to its cardiac counterpart, sMyBP-C also undergoes phosphorylation (Ackermann and Kontrogianni-Konstantopoulos, 2011a). Interestingly, while phosphorylation of cMyBP-C is restricted to the M-motif, phosphorylation of sMyBP-C takes place primarily in the Pro/Ala-rich motif and to a lesser extent in the M-motif (Ackermann and Kontrogianni-Konstantopoulos, 2011a). In particular, proteomics studies confirmed by the use of phospho-specific antibodies demonstrated that in the Pro/Ala-rich motif Ser-59 and Ser-62 are substrates of PKA, and Thr-84 is substrate of PKC, while in the M-motif Ser-204 is substrate of both PKA and PKC (Figure 1). Ser-59 and Ser-204 reside in alternatively spliced exons 5 and 10, respectively, and are therefore present in select slow variants (Ackermann and Kontrogianni-Konstantopoulos, 2013). Consistent with a purported important role of phosphorylation in the regulation of MyBP-C, the phosphorylation levels of sMyBP-C are differentially altered in relation to different (patho)physiological stressors. Accordingly, the phosphorylation levels of sMyBP-C are significantly reduced in fast-twitch Flexor Digitorum Brevis (FDB) muscle as a function of aging and dystrophy (Ackermann et al., 2015b). Similarly, the phosphorylation levels of sMyBP-C are notably decreased in slow-twitch soleus muscle as a result of aging and dystrophy, but increased in response to fatigue (Ackermann et al., 2015a). Although these observations are interesting, a detailed examination of the effects of individual or combinatorial phosphorylation events in the modulation of the structural and regulatory activities of sMyBP-C is currently lacking. It is therefore expected that future endeavors combining sophisticated *in vitro* and *in vivo* approaches will shed light on the role of phosphorylation in the modulation of sMyBP-C.

## *MYBPC1*: a recent myopathic gene

*MYBPC3* has garnered much attention over the past several decades due to its prevalent mutational rate leading to congenital hypertrophic and dilated cardiomyopathy (Harris et al., 2011; Santos et al., 2012; Kuster and Sadayappan, 2014; Lynch et al., 2015). It is only recently, however, that mutations in *MYBPC1* have been directly associated with inherited myopathies, and specifically with severe and lethal forms of distal arthrogryposis myopathy (Table 1) (Markus et al., 2012; Ha et al., 2013; Li et al., 2015). Contrary to *MYBPC3* mutations that mainly result in truncated proteins and function via haploinsufficiency (Marston et al., 2012; Kuster and Sadayappan, 2014; Barefield et al., 2015; Carrier et al., 2015), the currently known *MYBPC1* mutations have been suggested to result in poisonous proteins and manifest in a dominant negative manner after incorporation into sarcomeres (Markus et al., 2012; Ha et al., 2013; Li et al., 2015).

**Table 1:** **Current disease-causing mutations in ***MYBPC1*** and ***MYBPC2*****.

**Mutation**	**Gene**	**Domain**	**Inheritance**	**Organism**	**Phenotype**	**Disease**	**References**
W236R	*MYBPC1*	M-motif	Autosomal dominant	Human	Bilateral clubfoot, camptodactyly with ulnar deviations of the fingers, no facial weakness	Distal Arthrogryposis-1	Gurnett et al., 2010
Y856H	*MYBPC1*	C8	Autosomal dominant				
E359K	*MYBPC1*	C2	Autosomal dominant	Human	Ulnar deviations of the fingers, camptodactyly, overriding toes and planovalgus in lower limbs, facial anomalies including nasolabial folds and pouting/pinched lips, scoliosis	Distal Arthrogryposis-2	Li et al., 2015
P319L	*MYBPC1*	C2	Autosomal dominant				
R318STOP	*MYBPC1*	C2	Autosomal recessive	Human	Multiple joint contractures, micrognathia, anterior horn atrophy in spinal cord, fetal akinesia, postnatal lethal	Lethal Congenital Contractural Syndrome 4	Markus et al., 2012
L295R	*MYBPC1*	M-motif	Unknown	Bull	Muscle tremors, muscle weakness, inability to stand, ataxia, increased muscle tone, reduced spinal reflexes	Distal Arthrogryposis-1 like	Wiedemar et al., 2015
T236I	*MYBPC2*	M-motif	Compound heterozygote with *GPR126* homozygous R7STOP mutation	Human	Narrow thorax, polyhydramnios during development, and postnatal death	Unclassified Distal Arthrogryposis	Bayram et al., 2016
S255T	*MYBPC2*	M-motif					

Arthrogryposis, also known as arthrogryposis multiplex congenita, is clinically defined by congenital joint contractures or movement restriction in multiple body areas (Bayram et al., 2016). Generally, arthrogryposis occurs as a secondary effect of decreased fetal joint mobility, which can result from abnormalities of the central nervous system, the neuromuscular system, the skeletal system, or connective and cartilage tissue disturbances. (Filges and Hall, 2013; Haliloglu and Topaloglu, 2013; Hall, 2014; Bayram et al., 2016). Distal arthrogryposis (DA) myopathies are a group of autosomal dominant arthrogryposis disorders that mainly involve the distal parts of the limbs (Bamshad et al., 2009). Ten different types of DA have been described to date that share common general features, including a consistent pattern of hand and foot defects, limited involvement of proximal joints and variable expressivity (Bamshad et al., 1996; Krakowiak et al., 1998; Stevenson et al., 2006).

DA type-1 (DA-1) is the most common DA myopathy that affects approximately 1 in 10,000 individuals, and results in contractures limited to the distal muscles of the hands and feet. These include clubfoot, vertical talus, camptodactyly, overriding fingers and ulnar deviations of the fingers with no additional anomalies (Hall, 1985; Klemp and Hall, 1995; Gurnett et al., 2010). DA type-2 (DA-2) is a more severe form of DA, also characterized by contractures of the hands and feet, that is often accompanied by mild to severe craniofacial anomalies and/or scoliosis (Kulkarni et al., 2008; Bamshad et al., 2009). There are two subtypes of DA-2, including DA-2A (Freeman-Sheldon syndrome) and DA-2B (Sheldon-Hall syndrome) (Kulkarni et al., 2008; Bamshad et al., 2009; Li et al., 2015). While individuals with DA-2B Sheldon-Hall syndrome display mild to moderate facial contractures, individuals with DA-2A Freeman-Sheldon syndrome have moderate to severe facial contractures (Beck et al., 2013; Li et al., 2015).

In the last 5 years, dominant missense mutations in *MYBPC1* have been linked to the development of both DA-1 and DA-2 (Gurnett et al., 2010; Li et al., 2015). Specifically, missense mutations, W236R and Y856H, located in the M-motif and Ig domain C8, respectively, have been linked to DA-1 (Figure 1; Gurnett et al., 2010). Both of these substitutions are present in constitutively expressed exons and thus are contained in all slow variants (Gurnett et al., 2010; Ackermann et al., 2015b). ATPase staining of biopsies obtained from the distal Abductor Hallucis (AH) foot muscle of DA-1 patients carrying either mutation revealed severe type-I fiber atrophy, although localization of the mutant proteins was unaltered (Gurnett et al., 2010). *In vitro* binding and actin sliding assays demonstrated that the presence of the W236R and Y856H mutations markedly diminished the ability of the NH_2_ and COOH termini of sMyBP-C, respectively, to bind actin and myosin, and regulate the formation of actomyosin crossbridges (Ackermann et al., 2013). Examination of the expression levels of mutant sMyBP-C that contained the Y856H or the W236R mutation in human biopsies of AH or gastrocnemius muscles, respectively, revealed that the total amounts of the protein were significantly reduced in AH (~25%), but not gastrocnemius, muscle compared to controls (Ackermann et al., 2015b). Although puzzling, since both DA-1 mutations reside in constitutive exons, this finding is in agreement with the selective effects of DA-1 on distal muscles, and the lack of a myopathic phenotype in proximal muscles. Interestingly, the phosphorylation profile of mutant sMyBP-C containing the Y856H mutation was also altered in the affected AH muscle, whereas the phosphorylation profile of sMyBP-C carrying the W236R mutation was unchanged in gastrocnemius muscle (Ackermann et al., 2015b). Accordingly, use of a panel of phospho-specific antibodies and phos-tag gel electrophoresis revealed that mutant sMyBP-C harboring the Y856H mutation in AH muscle was phosphorylated at all four known residues, however the extent of phosphorylation was decreased by 30–70% for individual phospho-sites, compared to control tissue (Ackermann et al., 2015b).

Recently, two novel autosomal dominant missense mutations in Ig domain C2 of sMyBP-C, P319L and E359K, were linked to the development of DA-2 (Figure 1) (Li et al., 2015). Although a mechanistic understanding of the effect(s) of these mutations is still lacking, it is tempting to speculate that they may affect binding to the S2 portion of myosin and/or actin via induction of an unfavorable conformation (P319L) or altered electrostatic interactions (E359K). Future studies using a combination of biochemical, structural, biophysical and *in vivo* approaches will address these hypotheses.

In addition to DA-1 and DA-2, *MYBPC1* has been directly linked to the development of a neonatal lethal form of arthrogryposis myopathy, referred to as Lethal Congenital Contracture Syndrome type-4 (LCCS-4; Markus et al., 2012). Specifically, an autosomal recessive nonsense mutation in Ig domain C2 of sMyBP-C results in a premature stop codon at amino acid 318 (R318Stop; Figure 1). Given the recessive inheritance of LCCS-4, along with the absence of any phenotypic or functional abnormalities in the heterozygous carriers, it is highly likely that the R318Stop mutation results in loss of sMyBP-C rather than a poisonous truncated protein. Nevertheless, if the mutant protein is indeed expressed, it will lack domains C3-C10 downstream of Ig C2, which contain binding sites for LMM, titin and obscurin (Okagaki et al., 1993; Freiburg and Gautel, 1996; Ackermann et al., 2009).

In addition to mutations in the human *MYBPC1* gene that are associated with severe and lethal forms of DA, a new mutation in the bull *MYBPC1* gene was recently identified, too (Wiedemar et al., 2015). In particular, a 2-week old female calf presented with muscle tremors since birth, standing difficulty and reduced spinal reflexes (Wiedemar et al., 2015). Whole genome sequencing analysis revealed a *de novo* missense mutation, L295R, localized in the M-motif following the Ig domain C1, similar to the human W236R mutation. Although the phenotypic manifestation of the L295R mutation is reminiscent of DA-1, it is further accompanied by muscle tremors, which is indicative of a more complex and/or severe myopathy. At this time, a mechanistic understanding of the effects of the L295R mutation is lacking.

As novel mutations in *MYBPC1* are being identified in the mammalian genome underscoring its role in skeletal muscle (patho)physiology, it is worth mentioning that recently *MYBPC2*, encoding fMyBP-C, was also linked to an unclassified, neonatal lethal DA in the form of a compound heterozygote (Bayram et al., 2016). Specifically, a patient presenting with narrow thorax, polyhydramnios during fetal development, and neonatal death was found to possess two missense mutations in *MYBPC2*, T236I and S255T, located in the M-motif. The same patient also contained an R7Stop homozygous mutation in the *GPR126* gene, which encodes a G-protein coupled receptor that regulates neural, cardiac, and ear development (Patra et al., 2014; Bayram et al., 2016). Although mutations in *GPR126* have been linked with isolated arthrogryposis multiplex congenital (Ravenscroft et al., 2015), it is likely that the additional mutations in *MYBPC2* contributed to the postnatal lethality of the carrier due to accumulating anomalies in motor neurons and muscle structure and function (Bayram et al., 2016).

Although at the current time limited, the above studies clearly indicate that mutations in the genes that encode the skeletal MyBP-C proteins (and especially the slow isoform) are intimately associated with the development of severe and lethal myopathies. Obviously, the challenge now lies in deciphering the cell processes that are altered or compromised due to individual mutations using sophisticated and high resolution *in vitro* approaches and appropriate *in vivo* models.

## *In vivo* models of *MYBPC1*: perspectives and endeavors

Over the last four decades, a tremendous emphasis has been placed on the regulation and roles of cMyBP-C due to its direct involvement in congenital heart disease resulting in the generation of multiple animal models (Harris et al., 2002; Sadayappan et al., 2005; Carrier et al., 2015). This is not the case for sMyBP-C (or fMyBP-C). Remarkably though, the direct association of *MYBPC1* with the development of severe and lethal forms of DA has tunneled the interest of the scientific community toward the molecular and functional characterization of *MYBPC1*, too.

A recent study used the zebrafish model and antisense morpholinos to down-regulate the expression of *MYBPC1* (Ha et al., 2013). Knock-down zebrafish exhibited severe ventral body curvature, decreased mobility, and early lethality, along with impaired sarcomeric development and reduced number of myofibrils (Ha et al., 2013). Moreover, overexpression of mutant sMyBP-C proteins carrying either of the DA-1 mutations, W236R or Y856H, in zebrafish demonstrated that both mutant proteins exerted a dominant negative effect, resulting in embryos with mild bent body curvature, impaired mobility, and muscles with less tightly compacted fibers compared to controls (Ha et al., 2013).

Apart from the zebrafish model, no mammalian *MYBPC1* animal models have been generated yet. Our group has been systematically working on sMyBP-C for the last 5 to 6 years focusing on its molecular characterization, regulation and functional evaluation. Given the unique complexity of *MYBPC1* (Ackermann and Kontrogianni-Konstantopoulos, 2010, 2011b, 2013), its early expression during fetal development preceding that of *MYBPC2* (Gautel et al., 1998; Kurasawa et al., 1999), and the neonatal lethality of LCCS-4 patients, who most likely lack sMyBP-C, we predict that a constitutive *MYBPC1* null model would be postnatal lethal. If this is the case, such a model is still worth generating, since it will highlight the non-redundant roles of sMyBP-C and fMyBP-C, and will allow the study of the structural and regulatory roles of sMyBP-C in myofibrillar assembly and contractility during embryogenesis and (early) postnatal life. Obviously, conditional null models would circumvent the potential neonatal lethality of a constitutive *MYBPC1* knock-out allowing the detailed investigation of the roles of sMyBP-C in modulating actomyosin contractility in mature muscles. Along the same lines, knock-in models carrying the DA-1, DA-2, or LSSC-4 mutations are also lacking, limiting our understanding of the effects of the respective mutations to *in vitro* studies, which although informative, need to be accompanied by *in vivo* data.

Type II bacterial Clustered Regularly Interspaced Short Palindromic Repeats-associated protein Cas9 (CRISPR-Cas9) mediated genome editing has emerged as a powerful tool for genetic manipulation. Unlike small interfering RNAs (siRNAs) or short hairpin RNAs (shRNAs), the CRISPR-Cas9 system is able to knockout individual gene expression at the genomic level with minimal off-target effects (Zhang et al., 2014; Humphrey and Kasinski, 2015). Conversely, CRISPR/Cas9 technology is also being refined for generating knock-in models to recapitulate disease development (Chu et al., 2015; Tu et al., 2015). As the technology becomes increasingly popular, the generation of constitutive or conditional *MYBPC1* knock-out and knock-in mouse models should be feasible in a fairly short amount of time.

*In vivo* gene transfer (IVGT) followed by electroporation is also an efficient, non-viral method for gene delivery that has been successfully used by several groups (Gehl, 2003; Spanggaard et al., 2012; Hu et al., 2014). Although the mechanisms underlying DNA electrotransfer are not yet fully elucidated, it has been suggested that permeabilization of the cell membrane as well as electrophoretic migration of the DNA are involved (Mir et al., 1999; Bureau et al., 2000; Golzio et al., 2002; Satkauskas et al., 2002). Muscle tissue is a favorable target for gene electrotransfer as it is easily accessible, allowing high-level, long-term transgenic expression (Mir et al., 1999; Lucas and Heller, 2001; Spanggaard et al., 2012). Such an approach could therefore be employed to knock-down (via shRNA technology) or knock-out (via CRISPR-Cas9 technology) *MYBPC1* in a muscle-specific manner, circumventing the potential neonatal lethality of a constitutive null model, and enabling the functional examination of different slow variants given that they are expressed in distinct combinations among skeletal muscles. Moreover, IVGT combined with electroporation could be used to overexpress myopathic forms of sMyBP-C. Although the stoichiometry of endogenous to exogenous proteins is an important issue to consider, it could potentially be alleviated by inducible, titratable expression systems. Notably, IVGT experiments are particularly beneficial as the effects of gene knock-down, knock-out or knock-in experiments can be analyzed from the single fiber to the whole animal level. For instance, many groups have taken advantage of permeabilized muscle fiber preparations of human biopsies or pre-clinical mouse models to elucidate the (patho)physiology of cMyBP-C (Harris et al., 2002; Stelzer et al., 2006; James and Robbins, 2011; Wang et al., 2016).

An alternative to generating animal models of muscle disease has emerged in the last few years, entailing the development and propagation of grafts of myopathic or dystrophic human muscle tissue in mice (Riederer et al., 2012; Meng et al., 2014; Sakellariou et al., 2015). This approach has tremendous benefits, given that animal models often fail to replicate the features of human muscle disease. Along these lines, a recent study reported the generation of xenografts from human bicep muscle biopsies of facioscapulohumeral muscular dystrophy (FSHD) patients that were transplanted into the hindlimbs of immunodeficient NOD-*Rag1*^*null*^*IL2ry*^*n*^^*ull*^ mice (Sakellariou et al., 2015). The engrafted human muscle was efficiently regenerated and innervated, and displayed normal contractile properties (Sakellariou et al., 2015). While the xenografting model approach is still being perfected, the largest hurdle is the unavailability of fresh muscle biopsies and the lack of organized biobanks; obviously, this is a major issue that applies to *MYBPC1* related myopathies, as well. Nevertheless, the xenografting model could prove to be an extremely useful tool for studying the effects of human myopathies *in vivo*, since it may recapitulate the course of disease development more faithfully compared to engineered models of *C. elegans*, zebrafish, or mouse that are commonly used to date.

## Conclusions

Slow skeletal MyBP-C has recently attracted considerable interest primarily due to its direct involvement in the development of severe and lethal forms of distal arthrogryposis myopathy. Contrary to the fast and cardiac isoforms, sMyBP-C comprises a subfamily of proteins with possibly distinct structural and regulatory roles, which are modulated by constitutive and variant-specific phosphorylation events. Given the recent involvement of *MYBPC1* in severe and lethal myopathies, we predict that a comprehensive, multidisciplinary evaluation of its regulation and roles in health and disease is in order.

## Author contributions

JG and AK-K drafted, revised and approved the final version of the manuscript.

## Funding

This work was supported by NIH/NIAMS (Training Program in Muscle Biology, T32 AR007592-17 to JG), and the Muscular Dystrophy Association (Research Grant 313579 to AK-K).

### Conflict of interest statement

The authors declare that the research was conducted in the absence of any commercial or financial relationships that could be construed as a potential conflict of interest.
